# Specialization in habitat use by coral reef damselfishes and their susceptibility to habitat loss

**DOI:** 10.1002/ece3.321

**Published:** 2012-07-27

**Authors:** Morgan S Pratchett, Darren J Coker, Geoffrey P Jones, Philip L Munday

**Affiliations:** 1 ARC Centre of Excellence for Coral Reef Studies, James Cook UniversityTownsville, Queensland, Q4811, Australia; 2 School of Marine and Tropical Biology, James Cook UniversityTownsville, Queensland, Q4811, Australia

**Keywords:** *Acanthaster planci*, coral reef fishes, disturbance, ecological versatility, habitat degradation

## Abstract

While it is generally assumed that specialist species are more vulnerable to disturbance compared with generalist counterparts, this has rarely been tested in coastal marine ecosystems, which are increasingly subject to a wide range of natural and anthropogenic disturbances. Habitat specialists are expected to be more vulnerable to habitat loss because habitat availability exerts a greater limitation on population size, but it is also possible that specialist species may escape effects of disturbance if they use habitats that are generally resilient to disturbance. This study quantified specificity in use of different coral species by six coral-dwelling damselfishes (*Chromis viridis*, *C. atripectoralis*, *Dascyllus aruanus*, *D. reticulatus*, *Pomacentrus moluccensis*, and *P. amboinensis*) and related habitat specialization to proportional declines in their abundance following habitat degradation caused by outbreaks of the coral eating starfish, *Acanthaster planci*. The coral species preferred by most coral-dwelling damselfishes (e.g., *Pocillopora damicornis*) were frequently consumed by coral eating crown-of-thorns starfish, such that highly specialized damselfishes were disproportionately affected by coral depletion, despite using a narrower range of different coral species. Vulnerability of damselfishes to this disturbance was strongly correlated with both their reliance on corals and their degree of habitat specialization. Ongoing disturbances to coral reef ecosystems are expected, therefore, to lead to fundamental shifts in the community structure of fish communities where generalists are favored over highly specialist species.

## Introduction

Habitat degradation has a devastating influence on the structure and dynamics of ecological assemblages (Vitousek 1997; Fahrig 2001) and is increasingly recognized as the major contributor to global biodiversity loss (Brooks et al. 2002; Hoekstra et al. 2005). In general, habitat degradation results from depletion of key habitat-forming species (e.g., trees, kelp, corals) leading to declines in habitat-area and structural complexity, or increased habitat fragmentation (Caley et al. 2001; Alison 2004). The specific effects of disturbances on habitat-forming species, as well as habitat-associated species, depend on the frequency, severity, and selectivity of individual disturbances. Importantly, moderate disturbances may have highly selective effects and affect only a very limited suite of different species, but these moderate disturbances may nonetheless have very important influences on biodiversity and community structure (Connell 1978). Understanding species-specific responses to habitat degradation requires extensive knowledge of patterns of habitat use, including measures of habitat specialization, as well as knowledge of specific effects of disturbance on each habitat type (McKinney 1997). In general, habitat specialists are expected to be much more vulnerable to habitat degradation compared with species with generalized habitat requirements (Brown 1984; McKinney 1997; Vazquez and Simberloff 2002; Safi and Kerth 2004). However, it is also possible that specialist species may escape any effects from major disturbances because they use a relatively narrow range of resources, but this will only be true if they utilize habitats that are generally unaffected.

Coastal marine ecosystems are particularly susceptible to habitat degradation, where natural disturbances, climate change, and direct anthropogenic stresses have combined to cause extensive and widespread depletion of major habitat-forming species, including seagrasses, mangroves, and reef-building corals (Jackson et al. 2001; Steneck et al. 2002; Hughes et al. 2003). For coral reef ecosystems, habitat degradation is largely manifested as declines in the abundance of scleractinian corals (Hughes et al. 2003; Bellwood et al. 2004), which may be combined with increases in the abundance of alternative habitat-forming biota (e.g., macroalgae or soft corals). Globally, coral reefs are facing significant and accelerating coral loss (Gardner et al. 2003; Bellwood et al. 2004; Bruno and Selig 2007). Wilkinson (#b^200^) estimated that 20% of the world's coral reefs have already been “destroyed,” whereby coral cover has declined by >90% and there is limited prospect of recovery. Coral cover has also declined by 20–90% on a further 50% of the world's coral reefs, and these reefs may be “destroyed” by 2050 (Wilkinson #b^200^). Overall declines in the abundance of corals can have significant negative effects on coral reef fishes (Kaufman 1983; Dawson-Shepherd et al. 1992; Jones et al. 2004; Munday 2004; Wilson et al. 2006; Pratchett et al. 2008) and other motile reef organisms (Caley et al. 2001; Stella et al. 2011). However, the specific effects of coral reef degradation also depend greatly on the spatial and taxonomic extent of coral depletion (Pratchett et al. 2008).

Coral colonies represent distinct habitat units that are independently affected by disturbances, such as tropical storms, climate-induced coral bleaching, or infestations of the coral-feeding crown-of-thorns starfish, *Acanthaster planci* (Karlson and Hurd 1993; Hughes et al. 2003). Such disturbances tend to have a disproportionate impact on branching corals (Dollar and Tribble 1993; McClanahan et al. 2004), and thereby have important influences on both the biological and physical structure of coral reef habitats (Wilson et al. 2006, 2008). A significant proportion of coral reef fishes live very close to reef substrates and strongly associate with habitat structure provided by scleractinian corals; Jones et al. (2004) showed that up to 75% of coral reef fishes rely on live corals for food, shelter, or settlement habitat. However, there is considerable variation in the range of corals utilized by fishes, ranging from highly specialist fishes that are critically dependent on a single coral species (Munday 2004; Pratchett 2005a) to fishes that tend to utilize a range of different corals with broadly similar growth forms (Wilson et al. 2008).

Declines in the abundance of fishes following localized coral loss reflect the important role of live corals in providing biological and physical habitat for many reef fishes (Wilson et al. 2006). However, fishes that are first and worst affected by coral loss are those species with very strong dependence on corals (Munday et al. 2008) and are further reliant on only a very restricted set of available corals (Munday 2004; Feary 2007; Pratchett et al. 2008). Most notably, the loss of live coral cover leads to rapid and pronounced declines in abundance of highly specialized coral-feeding fishes (e.g., *Chaetodon trifascialis*), which are directly reliant on a very limited set of different corals for food (Pratchett et al. 2008). Similarly, there are many coral-dwelling fishes that highly specialized in their patterns of coral use. Munday (2004) showed that some species of gobies associate with just one or a few different coral species and that these fishes are facing extinction due to recent declines in the abundance of critical host corals. Following local depletion of preferred corals, coral-dependent fishes may persist by using generally nonpreferred corals (e.g., Pratchett et al. 2004), though this is likely to have significant consequences for individual fitness and survival (Munday 2001; Pratchett et al. 2006). Moreover, some highly specialized fishes appear incapable of using alternate corals (Berumen and Pratchett 2008).

While patterns of habitat use are key to predicting effects of habitat degradation on motile species (Feary et al. 2007; Wilson et al. 2008), there is surprisingly limited data on the range of resources (food and habitat) used by most coral reef fishes. The purpose of this study was to examine the range of coral species used by coral-dwelling damselfishes, test for changes in habitat use following coral depletion caused by *A. planci*, and establish whether species that are more specialized in their use of coral habitats have higher vulnerability to disturbance. Patterns of habitat use by coral-dwelling damselfish (specifically *Dascyllus aruanus* and *Pomacentrus moluccensis*) have been explored previously (e.g., Sale 1971, 1972; Holbrook et al. 2000). However, their degree of habitat specialization and their reliance on specific species of live corals is largely unknown. Sufficient evidence exists to suggest that coral-dwelling damselfishes have clear habitat preferences (Holbrook et al. 2000), but previous studies that explored variation in the vulnerability of damselfishes to coral loss used relatively crude estimates of habitat specialization, classifying coral habitats to growth form rather than species (e.g., Wilson et al. 2008). This study further tested for changes in abundance and patterns of habitat use by damselfishes during changes in habitat availability associated with infestations of *A. planci*. We hypothesized that proportional declines in the abundance of different damselfishes would correlate with their degree of habitat specialization, but this would depend on whether coral habitats used by specialized damselfishes are actually susceptible to predation by *A. planci*.

## Materials and Methods

This study was conducted at Lizard Island (14^o^40′S, 145^o^27′E), northern Great Barrier Reef, Australia. Underwater visual surveys were used to assess changes in abundance and microhabitat use of coral dwelling over 11 months (February 1998–January 1999), during a reef-wide outbreak of the coral-predator *A. planci* (Pratchett 2005b, 2010). Sampling was conducted at six locations along the fore-reef of Lizard Island (North Reef, Washing Machine, Coconut Beach, Lizard Head, South Island, South Bay), and at two locations in the lagoon (East Palfrey and Middle Lagoon). Densities of *A. planci* varied greatly among locations (Pratchett 2005b) corresponding with spatial differences in the extant of coral depletion recorded during 1996–1999 (Pratchett 2010). Ten replicate transects were run in each of two distinct reef zones (3–4 m depth on reef crest and 7–10 m depth at the bottom of the reef slope) at each of the ten locations to quantify availability of coral habitats and associated abundance of coral-dwelling damselfishes. Transects were 20-m long and 2-m wide (40 m^2^), orientated parallel to the reef crest, and run from a haphazardly selected starting point within each zone, at each location and in each year. Every scleractinian coral (including dead but intact coral colonies), located >50% within the transect area and with a maximum diameter greater than 10 cm, was identified to species. A total 12,062 distinct coral colonies, including 64 different coral species as well as algal-covered skeletons of dead branching corals, were surveyed during this study.

Having established the availability of potential coral habitats, we then quantified the number of damselfishes that sheltered within each coral colony, including both live (with any amount of live tissue) and dead coral colonies. Only the six most abundant damselfish species known to utilize live coral were selected and recorded, including *Chromis atripectoralis* Weland and Shultz 1951, *Chromis viridis* (Cuvier, 1830), *Dascyllus aruanus* (Linnaeus, 1978), *Dascyllus reticulatus* (Richardson, 1846), *Pomacentrus amboinensis* Bleeker, 1868, and *P. moluccensis* Bleeker, 1853. To accurately count damselfishes, divers moved 1–2 m away from occupied corals and counted fish as they emerged. Counts were repeated several times where there was any uncertainty, and whenever colonies contained more than ten individuals. The few damselfishes (<5% across all five species) that were not clearly associated with specific coral colonies were included in the total densities for each transect, but excluded from analyses of coral use.

Effects of coral depletion on the abundance of coral-dwelling damselfishes were assessed by comparing the overall abundance of each species between February 1998 and January 1999. Species-specific differences in the abundance of damselfishes were analyzed using ANOVA, testing for differences between zones (2 levels), among locations (10 levels), and between years (2 levels). Count data was log-transformed prior to analysis to improve homogeneity and normality, and bonferroni-corrected alpha levels were used to account for inflated Type-I error rates from running separate analyses for each species. The purpose was to test for changes in overall abundance of each damselfish species and relate this to observed coral loss. Declines in coral cover were restricted to 6/8 locations (North Reef, Washing Machine, Coconut Beach, Lizard Head, South Island, and South Bay), corresponding with the occurrence of *A. planci* (Pratchett 2010). In contrast, coral cover did not change (and actually increased slightly) at East Palfrey and Middle Lagoon, where *A. planci* were rarely if ever seen. Hereafter, locations are divided into those that were affected (North Reef, Washing Machine, Coconut Beach, Lizard Head, South Island, and South Bay) and unaffected (East Palfrey and Middle Lagoon). To test for changes in occupation rates by coral-dwelling damselfishes, χ^2^ homogeneity tests were used to compare the number of occupied versus unoccupied colonies of each coral species between years (1998 and 1999). Data were pooled across locations and zones to provide adequate cell counts.

Habitat associations of coral-dwelling damselfishes were analyzed using log-linear analyses, following Munday (2000), to test whether damselfishes used particular corals disproportionately to their availability, and whether patterns of habitat use were consistent among locations and between years. Habitat use was analyzed using sequential testing of increasingly complex models until there was no significant improvement in the goodness-of-fit statistic to find the simplest combination of factors that could account for observed patterns of habitat use (Table 1). Only 10 habitat categories (*Acropora divaricata, A. millepora*, *A. valida*, *Echinopora lamellosa*, *Pocillopora damicornis*, *P. eydouxi*, *Porites cylindrica*, *Seriatopora hystrix*, *Stylophora pistillata*, and dead branching corals) were used in the analysis to maximize statistical power, representing the most frequently used habitats across all damselfish species. To ensure independence of observations, analyses were based on the presence/absence of each pomacentrid species in each colony, rather than number of damselfishes per colony (see Thomas and Taylor 1990; Munday 2000). Data were pooled across replicate transects and zones to provide adequate cell counts.

**Table 1:** Log-linear models used to test patterns of habitat use (adapted from Munday 2000). Hierarchical models were tested sequentially until there was no further improvement in the fit of the model to the data. Two models were considered as alternative conditional models (3a and 3b) in the progression from model 2 → 4

Model	Factors included	Hypothesis tested
1	Site × year	Coral use is proportional to availability
2	Coral + site × year	Corals used disproportionately to availability and the pattern uniform among locations and years
3a	Coral × year + site × year	Corals used disproportionately to availability, but the pattern changes between years
3b	Coral × site + site × year	Corals used disproportionately to availability, but the pattern changes between locations
4	Coral × year + Coral × site + site × year	Corals used disproportionately to availability, but the pattern changes between locations and between years

## Results

### Patterns of coral use

**Figure 1:**
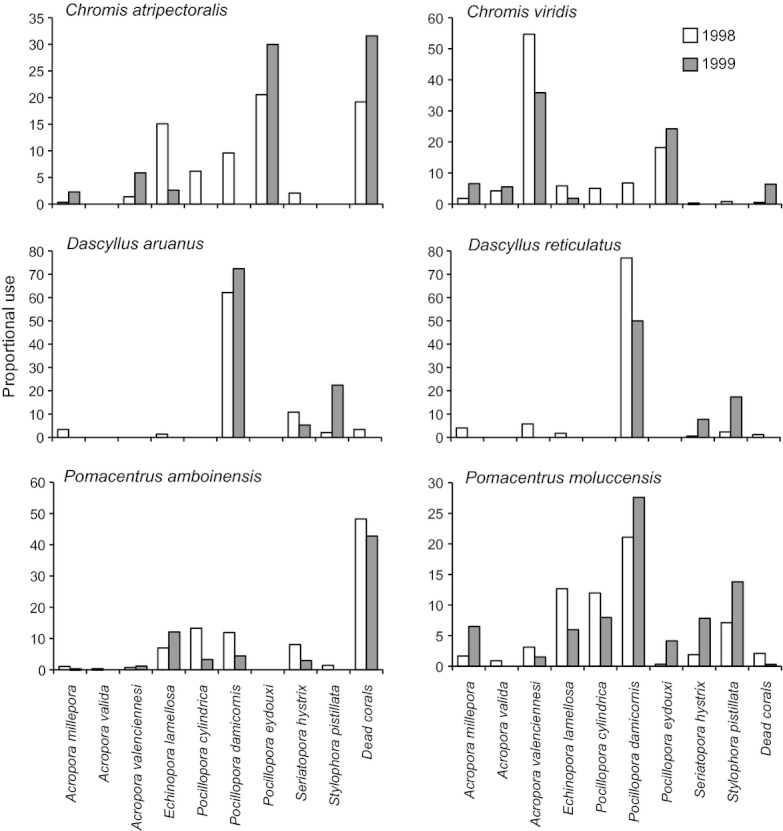
Proportional use of 10 habitat categories by each of the six species of damselfishes in 1998 and 1999. Based on log-linear analyses, only *Pomacentrus moluccensis* exhibited significant differences in patterns of habitat use between years.

A total of 8193 damselfishes (across all six species) were surveyed during the course of this study. Most damselfishes lived in close association with live coral colonies, although some individuals were found sheltering in algal-covered skeletons of dead branching corals. The proportion of individuals using dead but intact corals ranged from <1% for *D. reticulatus* (*n* = 226) to 48% for *P. amboinensis* (*n* = 675). For *C. viridis*, *D. aruanus*, *D. reticulatus*, and *P. moluccensis*, very few (<5%) individuals used dead corals (Fig. 1) and these species are hereafter referred to as obligate coral-dwelling species. In contrast, *C. atripectoralis* and *P. amboinensis* frequently use dead but intact coral skeletons and are thus considered to be facultative coral dwellers. Both *C. atripectoralis* and *P. amboinensis* used a relatively small subset of live coral taxa (15 and 18 coral taxa, respectively), but were much less dependent on individual coral colonies compared with *C. viridis*, *D. aruanus*, *D. reticulatus*, and *P. moluccensis*. Notably, *C. atripectoralis* and *P. amboinensis* often exhibited home ranges that encompassed several different coral colonies, and although they tended to use only large open branching corals (e.g., *Echinopora lamellose*, *Porites cylindrical*, and *Pocillopora eydouxi*), individual fishes would often alternate between two or more different coral colonies into which they sought shelter.

In February 1998, damselfishes used between 9 and 31 different habitat categories (including dead branching corals), but were predominantly found in just one or two different coral species. The most specialized species was *D. reticulatus* (Fig. 1), which was found living in only 8 different coral species. Moreover, 77% (134/174) of *D. reticulatus* were found in *P. damicornis*, which was also the predominant coral species used by *D. aruanus* and *P. moluccensis* (Fig. 1). *Chromis viridis* used markedly different coral species to the other coral-dwelling fishes and found predominantly on larger coral colonies of *P. eydouxi* and *A. valenciennesi*, but also used 18 other different coral species. *Pomacentrus moluccensis* was the least specialized of all obligate coral-dwelling damselfishes, using 30 different coral species including 18 different species of branching *Acropora*.

### Coral depletion and damselfish declines

**Figure 2:**
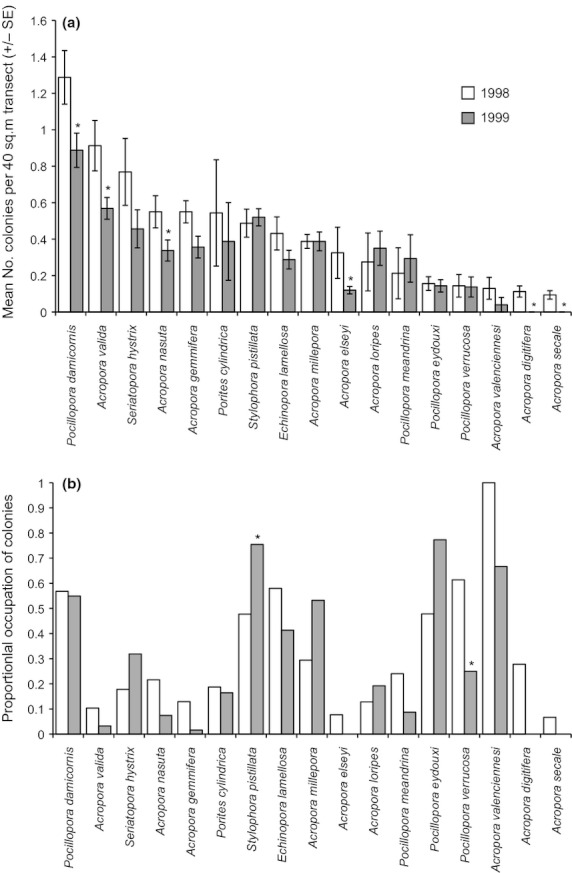
(a) Availability and (b) occupation (across all damselfishes) of predominant coral habitats used by coral-dwelling damselfishes at Lizard Island (northern Great Barrier Reef) in 1998 and 1999. “*” Indicates significant changes in availability and occupation between years.

Moderate declines (<20%) in overall cover of scleractinian corals occurred at Lizard Island during this study, from a mean of 8.2% cover (±0.6 SE) in February 1998, down to 6.6% cover (±0.5) in January 1999. Declines in coral cover were restricted to just the 6 (out of 8) locations, referred to as affected locations. Between zones, declines in the cover of scleractinian corals were most apparent on the reef slope, where coral cover declined from 10.3% cover (±0.7 SE) in February 1998, down to 7.1% cover (±0.4) in January 1999 (at affected locations). In contrast, average cover of corals on the reef crest actually increased over the same period. Declines in coral cover were further restricted to branching coral species, and mostly Acroporidae and Pocilloporidae corals. Significant declines in abundance were recorded for *P. damicornis*, *A. valida*, *A. nasuta*, and *A. esley*i, while *A. digitifera* and *A. secale* disappeared between 1998 and 1999 (Fig. 2). Total cover of *P. damicornis* declined by 45% from 0.77% cover (±0.14) in 1998 (data pooled across all zones and locations) down to 0.42% cover (±0.06) in 1999. Mean densities of *P. damicornis* colonies declined from 1.29 (±0.12) colonies per 200 m^2^ in February 1998 down to 0.89 (±0.14) colonies per 200 m^2^ in January 1999. However, *P. damicornis* was still the most abundant branching coral in 1999 (Fig. 2).

Declines in abundance of coral hosts led to corresponding declines in abundance of obligate coral-dwelling damselfishes (Table 2), despite relatively low occupation of most host corals (Fig. 2). Coral-dwelling damselfishes used only 7.6% (820/10,786 colonies) of potential coral hosts, and only 53% (34/64 species) of available coral species. Occupation rates were generally higher for Pocilloporidae (*Pocillopora*, *Seriatopora*, and *Stylophora*) corals than for most *Acropora* species, though 100% of colonies (7/7) of *A. valenciennesi* were occupied in 1998 (Fig. 2). Occupation rates of coral colonies were fairly consistent between years, and the only species for which significant changes in occupation were detected were *S. pistillata* and *P. verrucosa* (Fig. 2).

**Table 2:** Three-way ANOVA to explore variation in the abundance of each species of coral-dwelling damselfish

Species	Source	df	MS	*F*
*C. atripectoralis*	Year	1	0.19	0.17
	Zone	1	3.38	1.58
	Location	7	1.91	0.69
	Year × Zone	1	0.00	0.00
	Year × Loc.	7	1.16	2.15
	Zone × Loc.	7	2.14	3.97*
	Year × Zone × Loc.	7	0.54	0.80
*C. viridis*	Year	1	3.52	6.92*
	Zone	1	6.41	7.46*
	Location	7	1.31	2.79
	Year × Zone	1	2.21	2.46
	Year × Loc.	7	0.51	0.57
	Zone × Loc.	7	0.86	0.96
	Year × Zone × Loc.	7	0.90	0.69
*D. aruanus*	Year	1	16.20	0.92
	Zone	1	143.11	4.47
	Location	7	30.07	0.66
	Year × Zone	1	12.01	0.66
	Year × Loc.	7	17.53	0.97
	Zone × Loc.	7	31.98	1.77
	Year × Zone × Loc.	7	18.08	2.31*
*D. reticulatus*	Year	1	2.23	8.04*
	Zone	1	9.56	18.94***
	Location	7	0.71	1.41
	Year × Zone	1	2.19	7.83*
	Year × Loc.	7	0.28	0.99
	Zone × Loc.	7	0.50	1.80
	Year × Zone × Loc.	7	0.28	0.96
*P. amboinensis*	Year	1	0.02	0.04
	Zone	1	64.53	42.80***
	Location	7	1.99	1.13
	Year × Zone	1	0.08	0.64
	Year × Loc.	7	0.37	3.10
	Zone × Loc.	7	1.51	12.61***
	Year × Zone × Loc.	7	0.12	0.21
*P. moluccensis*	Year	1	4.55	3.85
	Zone	1	72.39	15.79**
	Location	7	17.31	6.63
	Year × Zone	1	8.03	2.55
	Year × Loc.	7	1.18	0.37
	Zone × Loc.	7	4.58	1.45
	Year × Zone × Loc.	7	3.15	2.66**

Significant effects (* *P* < 0.05, ** *P* < 0.01, *** *P* < 0.001).

**Figure 3:**
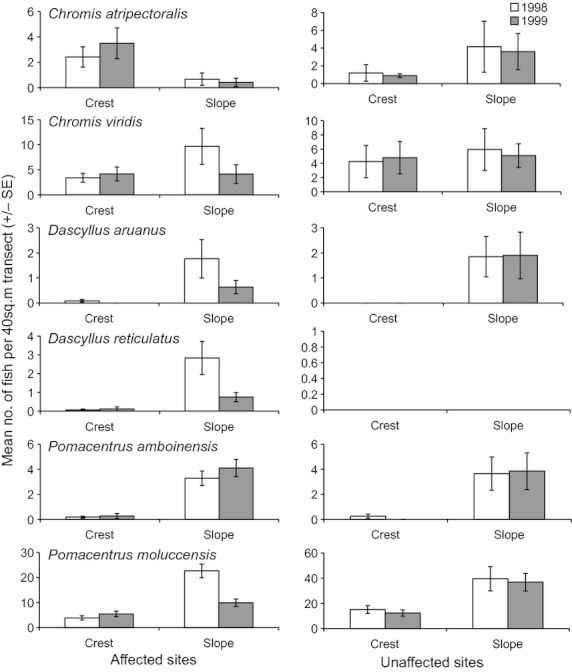
Mean abundance ± SE of damselfishes in crest and slope habitats at sites affected by outbreaks of *Acanthaster planci*, versus unaffected (natural control) sites in 1998 and 1999.

**Figure 4:**
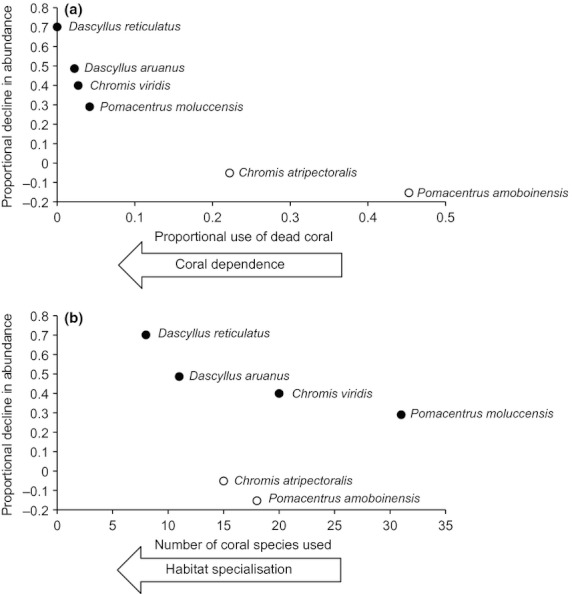
Proportional declines in the abundance of coral-dwelling damselfishes relative to (a) the proportional use of dead coral habitat (considered to be indicative of coral dependence), and (b) the number of different coral species actually used (as a measure of specialization). Damselfishes that are almost always found within live corals (obligate coral-dwelling species) are indicated by filled circles, whereas damselfishes that use live corals but frequently use dead coral habitats (facultative coral-dwelling species) are indicated by open circles. Data were pooled across all sites and across zones.

Temporal variation in abundance of obligate coral-dwelling damselfishes (*C. viridis*, *D. aruanus*, *D. reticulatus*, and *P. moluccensis*) varied between zones and/or among locations (Table 2). Declines in abundance were only apparent at fore-reef locations and also only on the reef slope (Fig. 3). The abundance of *C. atripectoralis* and *P. amboinensis* did not vary between years, but did differ among locations and between zones (Table 2). In addition to changes in overall abundance of some species (*C. viridis*, *D. aruanus*, *D. reticulatus*, and *P. moluccensis*), there were also changes in the density of damselfishes within remnant coral hosts. For *P. moluccensis*, the mean number of fish in occupied coral colonies increased slightly, from 6.61 (±0.51) fish per colony in 1998 to 7.03 (±0.32) fish per colony in 1999. However, the mean densities of all other fishes in occupied corals declined slightly between years. Overall declines in the abundance of coral-dwelling damselfishes corresponded to their use of specific coral habitats. More specifically, proportional declines in the abundance of the six damselfishes species were strongly correlated with their proportional use of live versus dead coral habitats, with coral-dependent species exhibiting much greater declines in abundance compared with those damselfishes that are considered to be facultative coral dwellers (*C. atripectoralis* and *P. amboinensis*). Also, among obligate coral-dwelling species (*C. viridis*, *D. aruanus*, *D. reticulatus*, and *P. moluccensis*), there was an apparent relationship between proportional declines in abundance and the number of different coral habitats actually used (Fig. 4). No formal analyses were undertaken to test the significance of these relationships due to the low number of species considered (six and four, respectively). However, it is obvious that there would be strong concordance with a line of best fit (although nonlinear) drawn through these data.

The predominant coral habitats used by coral-dwelling damselfishes (e.g., *P. damicornis*) varied greatly in their susceptibility to predation by *A. planci*. However, there was limited evidence of taxonomic shifts in patterns of coral use by most damselfishes. For five of the damselfish species (*C. atripectoralis*, *C. viridis*, *D. aruanus*, *D. freticulatus*, and *P. amboinensis*), the proportional use of different corals did not vary among locations or between years (Table 3). *Pomacentrus moluccensis* was the only species for which patterns of coral use significantly varied between years (Table 3) and diversity of habitat use actually contracted during the study. In 1998, *P. moluccensis* was seen living in 30 different coral species, but this dropped to 18 in 2009, following the localized depletion of several *Acropora* species (e.g., *A. aspera*, *A. cerialis*, *A. echinata*, *A. elseyi*, *A. humilis*, *A. intermedia*, *A. sarmentosa*, *A. tenuis* and *A. valida*). Corresponding with this decline in the use of most *Acropora* species, *P. moluccensis* increased its relative use *P. damicornis*, *S. hystrix*, and *A. millepora*, but did not use any coral species that were not used previously (in 1998).

**Table 3:** Log-linear analysis of habitat use by damselfish. Log-linear models (described in Table 1) were tested sequentially until there was no significant improvement in deviance (***P < 0.001; ns, nonsignificant)

Species	Model	Deviance	df	Improvement	df
*C. atripectoralis*	1	239.39	144		
	2	49.79	135	189.60***	9
	3	44.55	126	5.24 ns	9
	4	5.54	63	39.01 ns	63
*C. viridis*	1	293.39	144		
	2	39.79	135	253.60***	9
	3	34.05	126	5.74 ns	9
	4	5.54	63	28.51 ns	63
*D. aruanus*	1	402.56	144		
	2	45.98	135	356.58***	9
	3	30.16	126	15.82 ns	9
	4	0.55	63	29.61 ns	63
*D. reticulatus*	1	374.69	144		
	2	25.91	133	348.78***	11
	3	16.97	126	8.94 ns	7
	4	1.07	63	15.90 ns	63
*P. amboinensis*	1	362.67	144		
	2	85.46	133	277.21***	11
	3	68.57	126	16.89 ns	7
	4	20.56	63	48.01 ns	63
*P. moluccensis*	1	302.68	128		
	2	119.21	120	183.47***	8
	3a	74.91	112	44.30***	8
	4	12.74	56	62.17 ns	56

## Discussion

While it is generally assumed that specialist species are more vulnerable to disturbance compared to generalist counterparts (McKinney 1997), specialist species may escape effects of disturbance if they use habitats that are generally resilient to disturbance, especially given that specialist species, by definition, use a much narrower range of habitat types. It is also possible that seemingly specialist species increase their use of different resources following a disturbance, as a short-term mechanism to ameliorate effects of habitat loss (Pratchett et al. 2004). In this study, however, the predominant coral habitats used by coral-dwelling damselfishes (e.g., branching corals within the families Acroporidae and Pocilloporidae) were disproportionately depleted during localized infestations of *A. planci*, reflecting the known feeding preferences of this coral predator (e.g., Death and Moran 1998; Pratchett 2007). Consequently, high-specialized coral-dwelling damselfishes were disproportionately affected by coral depletion caused by localized infestations of *A. planci*. *Dascyllus reticulatus*, in particular, exhibited a 70% decline in abundance despite a very moderate (20%) decline in overall coral cover.

All six species of damselfishes considered in this study (*C. atripectoralis*, *C. viridis, D. aruanus*, *D. reticulatus*, *P. moluccensis*, and *P. amboinensis*) exhibited some degree of habitat specialization, using only corals with branching or digitate morphologies, as opposed to massive or plate-like corals (see also Ault and Johnson 1998a; Holbrook et al. 2000). However, *C. viridis*, *D. aruanus*, *D. reticulatus*, and *P. moluccensis* were the most specialized, exhibiting habitat specificity beyond the level of broadly defined coral morphologies (e.g., Wilson et al. 2008) and showing preference for specific coral species (mostly *Pocillopora* spp.). Moreover, patterns of habitat use were very consistent among locations and between years, despite significant differences in habitat availability and habitat composition. It is not surprising, therefore, that these damselfish species (*D. aruanus*, *D. reticulatus*, *C. viridis*, and *P. moluccensis*) experienced disproportionate declines during localized coral loss, whereas the abundance of more versatile species (*C. atripectoralis* and *P. moluccensis*) capable of using both live and dead coral were unchanged. While *P. amboinensis* is often found living on live corals, our data suggested that it is equally likely to associate with dead but intact corals. Similarly, Wilson et al. (2008) showed that 76% of *P. amboinensis* associate with dead coral habitats. Wilson et al. (2008) also showed that *C. atripectoralis* sometimes associates with dead coral habitats, although much less frequently than *P. amboinensis*. It is also apparent that *C. atripectoralis* is commonly observed to move between different areas of the reef and has low site fidelity. This low site fidelity obscures the reliance on specific coral types or live versus dead corals, but it also means that *C. atripectoralis* is likely to be affected by extensive coral loss across large areas of habitat rather than the selective removal of specific colonies. Recent outbreaks of *A. planci* at Lizard Island caused only relatively minor disturbance to benthic reef habitats compared with previous infestations of *A. planci* on the GBR (Pratchett 2010), or other major disturbances (Wilson et al. 2006; Pratchett et al. 2008). More severe disturbances are likely to have an even more pronounced effect on coral-dwelling damselfishes, with impacts extending to those species with comparatively weak reliance on live corals (Wilson et al. 2008).

Observed declines in the abundance of different coral-dwelling damselfishes were strongly associated with differences in their relative use of live (vs. dead) corals. Contrary to previous studies (e.g., Sano et al. 1984), this suggests that obligate coral-dwelling species (*D. aruanus*, *D. reticulatus*, *C. viridis*, and *P. moluccensis*) are strongly dependent on the biological habitat provided by live corals, as opposed to the physical structure of corals, which may be retained for several years following feeding activities of *A. planci*. Accordingly, *C. atripectoralis* and *P. amboinensis* may be much more affected by changes in structural complexity, rather than declines in coral cover. Experimental studies undertaken by Coker et al. (2009) showed that *D. aruanus* and *P. moluccensis* are much more susceptible to predation when associated with dead coral colonies, compared with live coral colonies, irrespective of changes in physical structure. It is likely therefore that observed declines in the abundance of these and other obligate coral-dwelling damselfishes relate to increased mortality rates, presumably due to predation. Declines in the abundance of coral-dwelling damselfishes may also be attributed to reduced availability of settlement habitat (Wilson et al. 2008), as many of these fishes are even more selective and heavily reliant on live corals as juveniles. Widespread reductions in coral cover may even reduce the recruitment success of *C. atripectoralis* and/or *P. amboinensis* (Wilson et al. 2008), even though there was no apparent effect on adult abundance. Alternatively, those fishes living on coral colonies consumed by *A. planci* may have moved to locations or habitats that were relatively unaffected by *A. planci* (*sensu* Wilson et al. 2006). We saw no corresponding increase in the abundance of these damselfishes at locations (within the lagoon) or zones (on the reef crest) where there were negligible densities of *A. planci* and no change in coral cover, but we cannot rule out movement of fishes to locations or habitats not surveyed.

Following resource depletion, animals might be expected to increase the range of resource types that they exploit (e.g., Devictor et al. 2010), thereby compensating for declines in the availability of preferred resources (e.g., Pratchett et al. 2004). This is, however, conditional upon species being functional specialists, as opposed to obligate specialists that are evolutionarily or behaviorally adapted to using a constrained set of alternative resources (Berumen and Pratchett 2008). For most of the species considered in this study, there was no change in their patterns of habitat use, which could reflect obligate specialization in the case of *C. viridis*, *D. aruanus*, and *D. reticulatus*, or limited susceptibility to the specific disturbance in the case of *C. atripectoralis* and *P. amboinensis*. The only species that did appear to change its patterns of habitat use was *P. moluccensis*, which actually used fewer different coral species in 2009, compared with 2008. This contraction in patterns of habitat use was caused by the localized depletion of many coral species (mostly, *Acropora* species) that were formerly used by *P. moluccensis*, although much less frequently than *P. damicornis*. *Pomacentrus moluccensis* escaped the worst effects of local coral depletion by using a wide range of different coral hosts that included both species that were highly susceptible and generally not susceptible to coral predation by *A. planci*. Clearly, declines in the abundance of all obligate coral-dwelling damselfishes would have been even more pronounced if they preferentially used *Acropora* coral species, many of which disappeared between 1998 and 1999.

Our findings add to an increasing number of studies from terrestrial and aquatic ecosystems showing that specialist species are more vulnerable to disturbance compared with generalist counterparts (e.g., nesting cavity specialization: Aitken and Martin 2008; habitat specialization: Kotze and O'Hara 2003; Fisher et al. 2003; dietary specialization: Charrette et al. 2006, Graham 2007; dietary and habitat specialization: Harcourt et al. 2002). The obvious question arising from these studies is what is the advantage conferred upon species that are ecologically specialized, especially when preferred resources are highly vulnerable to disturbance? For coral reef fishes, few studies have managed to show that the fitness of specialist species significantly exceeds that of generalist fishes when using common resources (e.g., Berumen and Pratchett 2008), whereas ongoing disturbances are leading to increasing abundance of generalist fishes, at the expense of many specialist species (Bellwood et al. 2006; Lawton et al. 2011). In terrestrial ecosystems (e.g., Kitahara and Fujii 1994; Novotny 1995), specialist species often dominate in relatively undisturbed environments but generalist species become increasingly abundant along gradients of human disturbance. This suggests that there may have been a fundamental shift in disturbance regimes on coral reefs, leading to increasing dominance of generalist fishes and motile invertebrates (Pratchett et al. 2008; Stella et al. 2011).

The degree of ecological specialization observed among coral-dwelling damselfishes is not atypical of coral reef fishes that recruit, shelter, and/or feed on corals (e.g., Kuwamurra et al. 1994; Munday et al. 1997; Munday 2002, 2004; Jones et al. 2004; Pratchett 2005a). Many species of *Gobiodon* (family Gobidae) are found in obligate association with just one or two different species of branching corals, mostly from the genus *Acropora* (Munday et al. 1997; Munday 2000). There are also strong parallels between preferred coral species for damselfishes, and those used by obligate coral dwellers of the genus *Paragobiodon* (Kuwamurra et al. 1994) and many crustacean symbionts (Knudsen 1967). Highly conserved patterns of habitat use suggest that certain corals, particularly *P. damicornis*, may offer selective advantages (e.g., increased survivorship) for coral-dwelling organisms. Consistent with this hypothesis, Jones (1988) and Beukers and Jones (1997) showed that survivorship of damselfishes (specifically *D. aruanus*, *P. amboinensis*, and *P. moluccensis*) was much higher in *P. damicornis* compared with other branching corals, such as *A. nobilis*. It is suggested that the morphological complexity of *P. damicornis* provides greater protection from predation compared with more simple or open branching patterns of other Pocilloporidae and Acroporidae corals (Beukers and Jones 1997). Even within coral species, certain colonies may be more favorable than others (Noonan et al. 2012). Habitat choice by obligate coral-dwelling damselfishes thus involves a trade-off between using the corals that maximize individual survivorship versus spreading risks associated with host coral mortality, using a range of different coral hosts that vary in susceptibility to major disturbances, which aids in the persistence of the species.

Results of this study add to the large body of evidence that reef fish populations (and assemblages) are highly structured according to the biological structure of benthic reef habitats (Ault and Johnson 1998b; Jones and Syms 1998). Although, the importance of habitat availability in determining the distribution and abundance of reef fish varies considerably among species (Munday and Jones 1998), extensive loss or degradation of coral reef habitats appears certain to reduce the abundance and diversity of coral reef fishes, especially highly specialized species (Jones et al. 2004; Wilson et al. 2006; Munday et al. 2008; Pratchett et al. 2008). This is the first study to demonstrate that obligate coral-dwelling damselfishes (particularly, *D. reticulatus*) that use only a limited range of coral species face a higher risk of extirpation and extinction compared with sympatric coral-dwelling (e.g., *P. moluccensis*) that utilize many different coral species. Observed declines in the abundance of obligate coral-dwelling damselfishes and their preferred habitats does not bode well for the future of these species given projected increases in the frequency, severity, and diversity of disturbances that are contributing to coral loss throughout the world (Gardner et al. 2003; Bellwood et al. 2004; Bruno and Selig 2007). However, the preferred coral habitats for coral-dwelling damselfishes (Acroporidae and Pocilloporidae) are also those corals that are likely to recover most rapidly in the aftermath of major disturbances (Linares et al. 2011), such that long-term persistence of these fishes will likely depend on their ability to recover and recolonize available corals following periodic disturbances. With increasing disturbance and habitat degradation, increased research is needed to assess overall resilience (not just vulnerability) of specialists versus generalists (*sensu* Hughes et al. 2003).

## References

[b1] Aitken KEH, Martin K (2008). Resource selection plasticity and community responses to experimental reduction of a critical resource. Ecology.

[b2] Alison G (2004). The influence of species diversity and stress intensity on community resistance and resilience. Ecol. Mongr.

[b3] Ault TR, Johnson CR (1998a). Relationships between habitat and recruitment of three species of damselfish (Pomacentridae) at Heron Reef, Great Barrier Reef. J. Exp. Mar. Biol. Ecol.

[b4] Ault TR, Johnson CR (1998b). Spatially and temporally predictable fish communities on coral reefs. Ecol. Mongr.

[b5] Bellwood DR, Hughes TP, Folke C, Nystrom M (2004). Confronting the coral reef crisis. Nature.

[b6] Bellwood DR, Hoey AS, Ackerman JL, Depczynski M (2006). Coral bleaching, reef fish community phase shifts and the resilience of coral reefs. Global Change Biol.

[b7] Berumen ML, Pratchett MS (2008). Trade-offs associated with dietary specialization in corallivorous butterflyfishes (Chaetodontidae: *Chaetodon*. Behav. Ecol. Sociobiol.

[b8] Beukers JS, Jones GP (1997). Habitat complexity modifies the impact of piscivores on a coral reef fish population. Oecologia.

[b9] Brooks TM, Mittermeier RA, Mittermeier CG, Rylands GAB, da Fonseca AB, Konstant WR (2002). Habitat loss and extinction in the hotspots of biodiversity. Cons. Biol.

[b10] Brown JH (1984). On the relationship between the abundance and distribution of species. Am. Nat.

[b11] Bruno JF, Selig ER (2007). Regional decline of coral cover in the Indo-Pacific: timing. Extent and subregional comparisons. PLoS One.

[b12] Caley MJ, Buckley KA, Jones GP (2001). Separating the effects of habitat fragmentation, degradation, and loss on coral commensals. Ecology.

[b101] Charrette NA, Cleary DFR, Mooers AO (2006). Range-restricted, specialist Bornean butterflies are less likely to recover from ENSO-induced disturbance. Ecology.

[b14] Coker DJ, Pratchett MS, Munday PL (2009). Coral bleaching and habitat degradation increases susceptibility to predation for coral-dwelling fishes. Behav. Ecol.

[b16] Connell JH (1978). Diversity in tropical rainforests and coral reefs. Science.

[b17] Dawson-Shepherd AR, Warwick RM, Clarke KR, Brown BE (1992). An analysis of fish community responses to coral mining in the Maldives. Environ. Biol. Fish.

[b18] Death G, Moran PJ (1998). Factors affecting the behaviour of crown-of-thorns starfish (*Acanthaster planci* L.) on the Great Barrier Reef: 2: feeding preferences. J. Exp. Mar. Biol. Ecol.

[b19] Devictor V, Clavel J, Julliard R, Lavergne S, Mouillot D, Thiller W (2010). Defining and measuring ecological specialization. J. Anim. Ecol.

[b20] Dollar SJ, Tribble GW (1993). Recurrent storm disturbance and recovery, a long-term study of coral communities in Hawaii. Coral Reefs.

[b21] Fahrig L (2001). How much habitat is enough?. Biol. Cons.

[b22] Feary DA (2007). The influence of resource specialization on the response of reef fish to coral disturbance. Mar. Biol.

[b23] Feary DA, Almany GR, McCormick MI, Jones GP (2007). Habitat choice, recruitment and the response of fishes to coral degradation. Oecologia.

[b24] Fisher DO, Blomberg SP, Owens IPF (2003). Extrinsic versus intrinsic factors in the decline and extinction of Australian marsupials. Proc. R. Soc. B.

[b26] Gardner TA, Côté IM, Gill JA, Grant A, Watkinson AR (2003). Long-term region-wide declines in Caribbean corals. Science.

[b27] Graham NAJ (2007). Ecological versatility and the decline of coral feeding fishes following climate driven coral mortality. Mar. Biol.

[b28] Harcourt AH, Coppeto SA, Parks SA (2002). Rarity, specialization and extinction in primates. J. Biogeog.

[b29] Hoekstra JM, Boucher TM, Ricketts TH, Roberts C (2005). Confronting the biome crisis: global disparities of habitat loss and protection. Ecol. Lett.

[b30] Holbrook SJ, Forrester GE, Schmitt RJ (2000). Spatial patterns in abundance of a damselfish reflect availability of suitable habitat. Oecologia.

[b31] Hughes TP, Baird AH, Bellwood DR, Card M, Connolly SR, Folke C (2003). Climate change, human impacts and the resilience of coral reefs. Science.

[b32] Jackson JBC, Kirby MX, Berger WH, Botsford LW, Bourque BJ, Bradbury RH (2001). Historical overfishing and the recent collapse of coastal ecosystems. Science.

[b33] Jones GP (1988). Experimental evaluation of the effects of habitat structure and competitive interactions on the juveniles of two coral reef fishes. J. Exp. Mar. Biol. Ecol.

[b34] Jones GP, Syms C (1998). Disturbance, habitat structure and the ecology of fishes on coral reefs. Aust. J. Ecol.

[b35] Jones GP, McCormick MI, Srinivasan M, Eagle JV (2004). Coral decline threatens fish biodiversity in marine reserves. Proc. Natl. Acad. Sci. USA.

[b36] Karlson RH, Hurd LE (1993). Disturbance, coral-reef communities, and changing ecological paradigms. Coral Reefs.

[b37] Kaufman LS (1983). Effects of hurrican Allen on reef fish assemblages near Discovery Bay, Jamica. Coral Reefs.

[b38] Kitahara M, Fujii K (1994). Biodiversity and community structure of temperate butterfly species within a gradient of human disturbance: an analysis based on the concept of generalist vs. specialist strategies. Res. Popul. Ecol.

[b39] Knudsen JW (1967). *Trapezia* and *Tetralia* (Decapoda, Brachyura, Xanthidae) as obligate ectoparasites of pocilloporid and acroporid corals. Pac. Sci.

[b40] Kotze DJ, O'Hara RB (2003). Species decline – but why? Explanations of carabid beetle (Coleoptera, Carabidae) declines in Europe. Oecologia.

[b41] Kuwamurra T, Yogo Y, Nakashima Y (1994). Population dynamics of goby *Paragobiodon echincephalus* and host coral *Stylophora pistillata*. Mar. Ecol. Prog. Ser.

[b42] Lawton RJ, Messmer V, Pratchettt MS, Bay LK (2011). High gene flow across large geographic scales reduces extinction risk for a highly specialised coral feeding butterflyfish. Mol. Ecol.

[b43] Linares C, Pratchett MS, Coker D (2011). Recolonisation and growth of *Acropora hyacinthus* following climate-induced coral bleaching on the Great Barrier Reef. Mar. Ecol. Prog. Ser.

[b44] McClanahan TR, Baird AH, Marshall PA, Toscano MA (2004). Comparing bleaching and mortality responses of hard corals between southern Kenya and the Great Barrier Reef, Australia. Mar. Poll. Bull.

[b45] McKinney ML (1997). Extinction vulnerability and selectivity: combining ecological and paleontological views. Annu. Rev. Ecol. Syst.

[b47] Munday PL (2000). Interactions between habitat use and patterns of abundance in fishes of the genus *Gobiodon*. Environ. Biol. Fish.

[b48] Munday PL (2001). Fitness consequences of habitat use and competition among coral-dwelling fishes. Oecologia.

[b49] Munday PL (2002). Does habitat availability determine geographical-scale abundances of coral-dwelling fishes?. Coral Reefs.

[b50] Munday PL (2004). Habitat loss, resource specialization, and extinction on coral reefs. Global Change Biol.

[b51] Munday PL, Jones GP (1998). The ecological implications of small body size among coral-reef fishes. Oceanogr. Mar. Biol. Annu. Rev.

[b52] Munday PL, Jones GP, Caley MJ (1997). Habitat specialisation and the distribution and abundance of gobies. Mar. Ecol. Prog. Ser.

[b53] Munday PL, Jones GP, Pratchett MS, Williams A (2008). Climate change and the future for coral reef fishes. Fish Fish.

[b54] Noonan SHC, Jones GP, Pratchett MS (2012). Coral size, health and structural complexity: effects on the ecology of a coral reef damselfish. Mar. Ecol. Prog. Ser.

[b55] Novotny V (1995). Reationshios between life histories of leafhoppers (Auchenorrhyncha-Hemiptera) and their host plants (Juncaceae, Cyperaceae, Poaceae). Oikos.

[b56] Pratchett MS (2005a). Dietary overlap among coral-feeding butterflyfishes (Chaetodontidae) at Lizard Island, northern Great Barrier Reef. Mar. Biol.

[b57] Pratchett MS (2005b). Dynamics of an outbreak population of *Acanthaster planci* at Lizard Island, northern Great Barrier Reef (1995–1999). Coral Reefs.

[b58] Pratchett MS (2007). Feeding preferences of *Acanthaster planci* (L.) under controlled conditions of food availability. Pac. Sci.

[b59] Pratchett MS (2010). Changes in coral communities during an outbreak of *Acanthaster planci* at Lizard Island, northern Great Barrier Reef (1995–1999). Coral Reefs.

[b60] Pratchett MS, Wilson SK, Berumen ML, McCormick MI (2004). Sublethal effects of coral bleaching on an obligate coral feeding butterflyfish. Coral Reefs.

[b61] Pratchett MS, Wilson SK, Baird AH (2006). Declines in the abundance of *Chaetodon* butterflyfishes (Chaetodontidae) following extensive coral depletion. J. Fish Biol.

[b62] Pratchett MS, Munday PL, Wilson SK, Graham NAJ, Cinner JE, Bellwood DR (2008). Effects of climate-induced coral bleaching on coral-reef fishes, ecological and economic consequences. Oceanogr. Mar. Biol. Annu. Rev.

[b63] Safi K, Kerth G (2004). A comparative analysis of specialization and extinction risk in temperate-zone bats. Cons. Biol.

[b64] Sale PF (1971). Extremely limited home range in a coral reef fish, *Dascyllus aruanus* (Pisces; Pomacentridae). Copeia.

[b65] Sale PF (1972). Influence of corals on the dispersion of the pomacentrid fish, *Dascyllus aruanus*. Ecology.

[b67] Sano M, Shimizu M, Nose Y (1984). Changes in structure of coral reef fish communities by destruction of hermatypic corals: observational and experimental views. Pac. Sci.

[b68] Stella JS, Pratchett MS, Hutchings PA, Jones GP (2011). Diversity, importance and vulnerability of coral-associated invertebrates. Oceanogr. Mar. Biol. Annu. Rev.

[b69] Steneck RS, Graham MH, Bourque BJ, Corbett D, Erlandson JM, Estes JA (2002). Kelp forest ecosystems, biodiversity, stability, resilience and future. Environ. Cons.

[b70] Thomas DL, Taylor EJ (1990). Study designs and tests for comparing resource use and availability. J. Wildlife Manage.

[b71] Vazquez DP, Simberloff D (2002). Ecological specialization and susceptibility to disturbance, conjectures and refutations. Am. Nat.

[b72] Vitousek PM (1997). Human-domination of Earth's ecosystems. Science.

[b200] Wilkinson C (2004). Status of coral reefs of the world: 2004.

[b73] Wilson SK, Graham NAJ, Pratchett MS, Jones GP, Polunin NVC (2006). Multiple disturbances and the global degradation of coral reefs: are reef fishes at risk or resilient?. Global Change Biol.

[b74] Wilson SK, Burgess SC, Cheal AJ, Fisher R, Miller I, Polunin NVC (2008). Habitat utilization by coral reef fish, implications for specialists vs. generalists in a changing environment. J. Anim. Ecol.

